# Fibrous dysplasia of the proximal femur: surgical management options and outcomes

**DOI:** 10.1007/s11832-014-0625-9

**Published:** 2014-11-20

**Authors:** Indranil V. Kushare, Dino Colo, Hooman Bakhshi, John P. Dormans

**Affiliations:** Division of Orthopaedic Surgery, The Children’s Hospital of Philadelphia, 3400 Civic Center Boulevard, Philadelphia, PA 19104 USA

**Keywords:** Fibrous dysplasia, Shepherd crook deformity, McCune-Albright syndrome

## Abstract

**Purpose:**

The management of proximal femoral deformity in fibrous dysplasia (FD) is a challenge to the orthopaedic surgeon. The purpose of this study was to analyze the various presentations of FD of proximal femur and the results of the various treatment modalities for the same.

**Methods:**

This is a retrospective cohort study of 23 patients (24 femora) with FD who underwent surgery for the proximal femur. The study sample included 14 males, nine females. Ten patients had a monostotic disease, eight patients had polyostotic disease, and five patients had McCune-Albright syndrome.

**Results:**

Group 1: shepherd crook deformity—included five patients who underwent femoral neck osteotomy. Four patients had intramedullary (IM) nailing with neck cross-pinning and all patients showed union. One patient was stabilized with external fixation, which failed. Group 2: nine patients (ten femora) presented with frank pathological fracture. Nine underwent fixation with IM nailing, one with locking plate and screws. Three patients had to undergo more than one procedure and all fractures showed good union. Group 3: nine patients who presented with bone cyst and pain. All patients underwent biopsy; four of them had curettage with bone graft.

**Conclusion:**

Shepherd crook deformity can be treated by a well-planned osteotomy and fixation with intramedullary implants with neck cross-pinning. Frank pathological fractures fixation with an intramedullary nail has excellent results even if not accompanied by resolution of the fibrodysplastic lesion. More than one procedure may be required. External fixation is not an optimal choice for fixation of femoral osteotomies in FD.

## Introduction

Fibrous dysplasia (FD) is a condition characterized by the presence of fibro-osseous tissue in the bone leading to widening and thinning of its cortex. Fibrous dysplasia has a wide clinical spectrum, with substantial variation between patients in terms of orthopaedic manifestations, including the number of fractures and the degree of deformity [1].

Fibrous dysplasia of the femoral neck is difficult to treat due to the varied presentations like pain, pathological fractures, severe deformity, and high chances of recurrence [2].

Clear guidelines for orthopaedic management of fibrous dysplasia in the proximal femur have not been established. The decision to manage a case of fibrous dysplasia of the proximal femur, especially shepherd crook deformity (bowing and varus deformity of the femoral neck region), is usually decided on a case-by-case basis by individual surgeons.

The aim of our paper was to analyze the various presentations of fibrous dysplasia of the proximal femur like pain, fractures, shepherd crook deformity, and describe the results of the various treatment modalities for the same.

## Patients and methods

After obtaining approval of the institutional review board, a retrospective analysis was done of 23 consecutive patients (24 femora) who underwent surgery at our institution for fibrous dysplasia of the proximal femur and subtrochanteric region from 2001 to 2012. The patients were included only if there was histological confirmation of fibrous dysplasia. There were 14 males (14 femora) and nine females (with ten affected femora). Out of 23 patients, ten had a monostotic disease, eight patients had polyostotic disease, and five patients had McCune-Albright syndrome (MAS) (Table 1).

**Table 1:** Demographics, clinical presentation, and treatment of patients with fibrous dysplasia

Type of FD	Case	Sex (M:F)	Group	Clinical presentation	Age at surgery (years)	Treatment	Need for further treatment	Pain at last follow-up
Monostotic	Case 4	M	3	R hip pain	6.2	IM nail	–	None
	Case 7	M	2	R femur Fx	13.1	IM nail	–	None
	Case 8	M	1	R hip pain, limp	14.6	IM nail	–	Pain from bursitis over the screw heads
	Case 10	M	2	R femur Fx	14.6	IM nail	–	None
	Case 13	M	2	R femur Fx	13.1	IM nail	–	None
	Case 15	M	3	R thigh pain	10.3	Curettage + bone graft	–	None
	Case 16	M	3	R hip pain	10.1	None	–	None
	Case 18	M	2	R femur Fx	5.4	IM nail	–	Pain
	Case 19	M	3	Incidental^a^	7.1	IM nail	–	None
	Case 21	F	3	R hip pain	14.3	Curettage + bone graft	–	
Polyostotic	Case 1	F	1	L hip pain, LLD	12.3	Osteotomy + ExFix	Fixation failure, hardware removal	
	Case 5	M	1	R hip pain	11.1	IM nail	–	
	Case 6	M	3	R hip pain	13.7	Osteotomy + Ex Fix	The fixator was removed in six months because of infection	None
	Case 11	F	2	L femur Fx	12.1	IM nail	–	Pain^b^
	Case 17	M	3	R hip pain	11.7	IM nail	–	None
	Case 20	F	2	L femur fracture	11.1	Initially with Spica cast followed by IM nail in two weeks	–	None
				LLD	12.7	R distal femoral and proximal tibial epiphysiodesis	–	
	Case 22	F	3	L hip pain	20	Curettage + bone graft	–	Osteomyelitis treated with I/D
	Case 23	F	1	L hip pain	16.7	Plate and screws	–	
MAS	Case 2	F	2	R femur Fx	12.7	IM nail	Yes—proximal femur osteotomy	None
				L femur Fx	15	IM nail	–	
	Case 3	F	1	R Femur Fx	17.2	IM nail	–	Minimal, limited ROM due to HO
	Case 9	M	2	R femur Fx	9.2	Locking plate and screws	–	
	Case 12	F	3	L hip pain	12	IM nail	–	Minimal
	Case 14	M	2	L femur Fx	11.4	IM nail	–	None

*FD* fibrous dysplasia, *MAS* McCune-Albright Syndrome, *R* right, *L* left, *Fx* fracture, *LLD* limb length discrepancy, *ExFix* external fixator, *IM* nail intramedullary nail, *ROM* range of motion, *HO* heterotopic ossification, *I/D* irrigation and debridement

^a^ This patient sustained left femur subtrochanteric fracture three weeks after open surgical curettage of the bone lesion and was subsequently treated surgically with IM nail

^b^ This patient had recurrent pain due to suspected microfracture

We divided our patient cohort into three groups according to presentation with either shepherd’s crook deformity, having increased bowing of the proximal femur and varus deformity of the femoral neck (Group 1)—five patients, a frank displaced pathological fracture through the fibrodysplastic lesion (Group 2)—nine patients, or bony lytic lesion with pain (Group 3)—nine patients.

Patient charts were evaluated for number and type of surgical procedures, recurrence, refracture, and presence of pain. Radiographic evaluation included union of the osteotomy, change in neck shaft angles for patients treated for shepherd’s crook deformity measured on an anteroposterior radiograph preoperatively and postoperatively.

The clinical outcomes were evaluated as satisfactory (mild or no pain, mild or no limp with activity causing no functional disability) and unsatisfactory (moderate/severe pain and severe limp with activity inferring with function). The choice of implant used for fixation after the surgery was noted.

The average age at the time of the index surgery was 12.29 (5–20) years. The average follow-up was 3.04 (1.17–6.45) years.

## Results

**Fig. 1:**
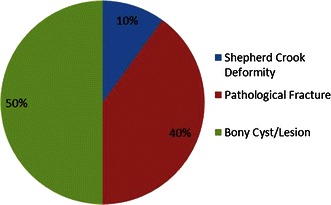
The types of presentation of proximal femoral deformity in patients with fibrous dysplasia

**Fig. 2:**
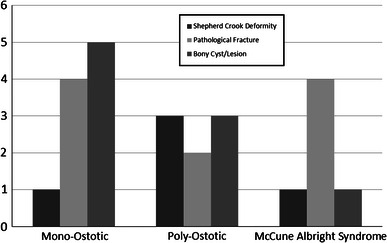
The distribution of fibrous dysplasia types between clinical presentation types

The patients with fibrous dysplasia were in three main clinical presentation groups: shepherd’s crook deformity, pathologic fracture, and bone lytic lesion with pain (Fig. 1; Table 2). The distribution of fibrous dysplasia types between clinical presentation types is demonstrated in Fig. 2.

**Table 2:** The patient sample categorized based on the type of fibrous dysplasia

	No. of patients	No. of femora	Sex (M:F)	Presentation			Age (years)	Total no. of surgeries	Follow Up (years)	Clinical outcomes	
				Shepherd crook deformity	Pathological fracture	Bony cyst/lesion				Satisfactory	Unsatisfactory
Monostotic	10	10	9:1	1	4	5	10.88	12	2.62	8	2
Polyostotic	8	8	3:5	3	2	3	13.57	12	3.83	7	1
McCune-Albright Syndrome	5	6	2:3	1	4	1	12.92	6	2.67	5	1

In the eight patients with polyostotic disease, four patients were type 6, three were type 1 and one was type 4 according to radiological classification by Ippolito et al.

**Fig. 3:**
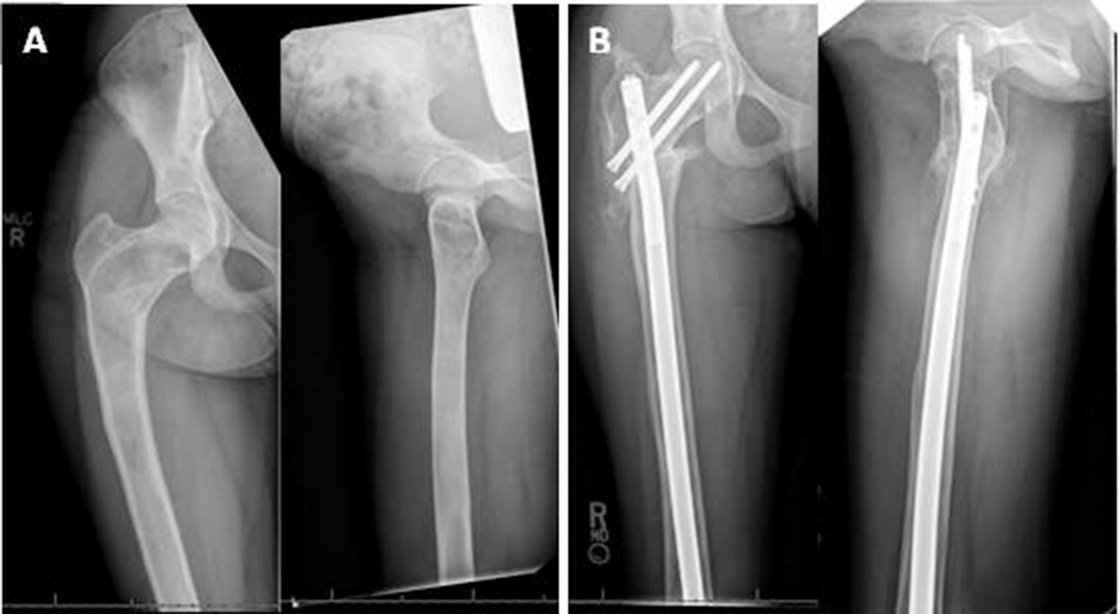
Seventeen-years-old female with McCune-Albright syndrome with shepherd’s crook deformity (**a**). The deformity was treated with osteotomy and intramedullary fixation (**b**)

Group 1: shepherd’s crook deformity: five patients presented with pain at the affected hip joint and a limp caused by a shepherd crook deformity (Fig. 3a). The average age of the patients at the time of surgery was 14.3 (12.3–17.2) years. Three patients had polyostotic disease, one had monostotic disease, and one had MAS. All of the patients underwent corrective osteotomies. The choice of implant used for fixation varied amongst patients according to the location of the lesion and quality of the bone. One patient treated with an osteotomy with external fixation, had loosening of the hardware, which had to be removed. This patient refused any further surgery. Amongst the remaining four patients, two underwent valgus osteotomy, one underwent multiple osteotomies, and one underwent a medial displacement osteotomy. Three patients had intramedullary (IM) nailing with neck cross-pinning (Fig. 3b) and one had a plate and screw fixation with a spica cast. Average neck shaft angle was increased from a preoperative 108° (98°–118°) to a postoperative 120.5° (100°–129°). Average follow-up for these patients was 2.2 (1.6–6.4) years. At the final follow-up, two patients had restriction in hip range of motion, one had a decrease in abduction and the other patient hade internal rotation, and two patients had persistent pain. All osteotomy sites showed complete union. The result was satisfactory in three patients and unsatisfactory in two patients.

**Fig. 4:**
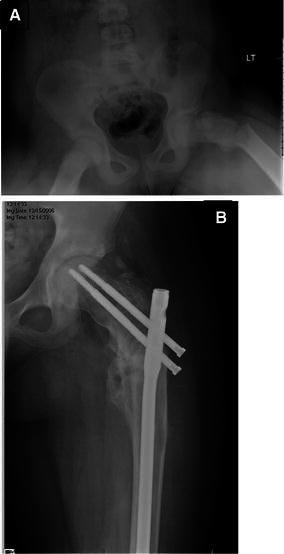
Subtrochanteric fracture of femur due to fibrous dysplasia (**a**). The fracture was treated with intramedullary nail and showed appropriate healing at latest follow-up (**b**)

Group 2: macroscopic pathological fractures for nine patients (ten femora) presented with frank pathological fractures. Of these, five were located in the proximal femur (Fig. 4) and five were subtrochanteric. Four patients were diagnosed with monostotic disease, two patients had polyostotic disease, and three patients had MAS. The average age was 11.77 (5.43–14.97) years at surgery. Eight patients underwent surgery with IM nailing as the primary method of treatment. One patient had a locking osteotomy plate inserted along with an incisional biopsy. One patient underwent closed reduction with a spica cast initially, followed by open reduction with an IM nailing. Three patients were required to undergo more than one procedure. Average follow-up was three (1.3–5.7) years. Results were satisfactory in nine out of ten patients. In one patient, the lesion progressed to develop into a shepherd’s crook deformity despite IM nailing being done for the fracture.

All fractures showed good union and there were no refractures or infections.

Group 3: cystic/lytic bone lesion with pain in nine patients presenting with hip pain were found to have a bony lesion on plain radiographs. Five patients were diagnosed with monostotic disease, three had polyostotic disease and one had MAS. Average age of these patients at the time of surgery was 11.7 (6.1–19) years. Four patients underwent curettage, three patients underwent a biopsy, one patient was followed by a partial diagnostic excision, one patient was combined with IM nailing, and one patient with curettage. Two patients underwent an osteotomy. The average time for follow-up was three (1.5–5.4) years. The result was satisfactory in all the patients in this group.

In the eight patients with polyostotic disease, four patients were type 6, three patients were type 1, and one patient was type 4 according to radiological classification by Ippolito et al. [3].

Three patients with multiple painful lesions were given bisphosphonates, and all of them had resolution of pain after therapy.

Complications: out of 23 patients who underwent various surgical procedures for the proximal femur, there were five complications. Two patients were treated with osteotomy and external fixator, one patient developed loosening of the pins, and the other patient developed infection at the pin sites. Both of the external fixations were removed within six months. One patient had pain from bursitis caused by the screw heads, one patient had heterotopic ossification at the insertion site of the nail causing decreased hip range of motion, and there was one case of postoperative hemorrhage followed by infection and osteomyelitis, which resolved with repeated debridement and antibiotics. The grading of the severity of the complications according to Goslings et al. [4] was grade 1(temporary disadvantage, no reoperation) for one patient, grade 2 (recovery after reoperation) for three patients, and grade 3 (probably permanent disability) for one patient.

## Discussion

FD is an orthopedic condition with a wide spectrum of presentation. The treatment of the dysplastic lesions in the proximal femur region is still somewhat unclear, and varies widely. Fibrous dysplasia of the bone can present as three clinical forms: monostotic, polyostotic and as a part of a McCune-Albright syndrome.

Out of the ten monostotic patients in our series, only one presented with shepherd crook deformity, four had significantly displaced pathologic fractures, and five had a painful lesion. Average age at surgery was 11.4 years as compared to 13.5 years in the polyostotic group. Patient age is important as monostotic lesions remain active only until skeletal maturity whereas polyostotic lesions may progress even during adulthood [1]. Two of the patients required an additional procedure and 80 % of the patients had a satisfactory result overall. These findings concur with the literature that monostotic lesions are easier to treat, have good outcomes, and require fewer number of procedures [2]. One of the patients that presented with a bony lytic lesion with pain had undergone only curettage and biopsy for diagnosis. The same patient suffered a pathologic fracture four weeks later and had to undergo intramedullary nailing for the same.

Out of the eight patients with polyostotic disease, four patients were type 6, three patients were type 1, and one patient was type 4 according to radiological classification by Ippolito et al. [3]. Three of these patients had shepherd crook deformity and two patients had to undergo three or more procedures. Our findings are similar to the series by Guille et al. [2] where they found that polyostotic involvement tended to need multiple surgical procedures. The need for multiple surgeries seems to be a result of poor bone quality, thus, leading to weak fixation of the implant in the sick bone compounded by the progressive nature of the disease.

The patients with multiple site involvement were referred for a metabolic consult in our institution. The reason was to rule out any MAS or other endocrine abnormalities that may directly affect bone turnover and bone mass even within a fibrous dysplastic bone segment [5]. Orthopaedic opinion might be the first consult for these patients, and it is recommended that there should be a thorough medical evaluation at the time of diagnosis [5]. When polyostotic disease is suspected, an endocrinology consult is recommended so that the associated endocrine abnormalities can be treated adequately [1, 6].

The expected functional outcomes in patients with MAS were poor overall [7]. Bone pain is very common with MAS [8]. Bisphosphonates relieved pain in three symptomatic patients with multiple lesions in our cohort. Bisphosphonates have been shown to offer pain relief in appropriately selected patients with fibrous dysplasia [1]. However, it has been suggested not to change the natural history of the disease [6].

Intramedullary nailing was used in 14 patients in our series and is a good fixation option for proximal femur, especially subtrochanteric fractures [9, 10]. They provide long-term stabilization of the femora and prevent refractures [5]. Long intramedullary nailing that traverses the neck and has a firm purchase in the head can prevent loss of subsequent neck shaft angle [11]. The prevention of loss of femoral neck shaft angle is important as it appears to have great effect on functional activity in children [12]. Elastic nails were used in one patient in our series who was 5.4 years old at the time of surgery. These elastic nails were replaced by an intramedullary rod at a later date due to their migration. Another patient who had curettage biopsy and grafting at age seven-years-old had a fracture at the surgical site and had to be treated by intramedullary implant. Earlier onset of symptoms can be indicative of more severe disease and involvement [7]. Even though intramedullary implants are the choice for implants, there are limitations to their use in younger patients [6]. Elastic nails or Ender nails do not prevent fractures or deformities and should not be routinely used. Their use should be limited to very young patients and should be replaced by cephalomedullary nails as soon as allowed by the femur size [5].

Shepherd crook deformity with bowing of the proximal femur is the pathognomonic lesion in FD [6]. Osteotomies, intramedullary nailing, and neck cross-pinning are the preferred methods for the management shepherd crook deformity [11]. Our limited data concurs with the same. Regarding the timing of the surgery, we recommend that the natural history of the deformity must be defined by the surgeon. The radiological classification by Ippolito et al. [3] can be helpful in polyostotic cases to determine the progressive deformities. We believe that earlier surgery is better if the deformity is progressive. The osteotomies could be valgus with medial displacing osteotomy [2] or multiple osteotomies [11]. In their study on 14 femora with shepherd’s crook deformity, Yang et al. [13] obtained satisfactory results with valgus osteotomy, curettage, massive impaction allograft, and intramedullary nail with neck cross-pinning. Li et al. [14] have shown that valgus osteotomy with dynamic hip screw internal fixation can give good results and improve function. Additionally, since both the patients with external fixators had to get them removed, it was found that external fixation is not an appropriate method of fixation in patients with fibrous dysplasia, and their use is not recommended.

The femoral neck was not stabilized and caused loss of neck shaft angle in five cases in the study by O’Sullivan et al. [8] and in two cases by Jung et al. [11]. Thus, stabilization of the femoral neck is crucial to prevent deformity progression.

In our series, we used a bone graft in eight out of 23 patients. The various graft material combinations that were used included autologous bone marrow, demineralized bone matrix, cancellous autograft, allograft, and medical grade calcium sulfate pellets. None of the 23 patients had complete radiographic resolution of the fibrodysplastic lesion, thus, questioning the role of curettage and bone grafting. In their series of treatment of 22 patients having fibrous dysplasia of the proximal femur, Guille et al. [2] reported that no lesion decreased in size following curettage and bone grafting. Cortical bone grafts are frequently resorbed and do not halt disease progression of deformity [5, 6].

Out of the five patients in our study who had diagnostic biopsy and curettage for monostotic lesion, one sustained a fracture after 27 days after the initial procedure and was managed with intramedullary femur nailing. Simple curettage or curettage with autogenous cancellous bone graft has high recurrence [1]. Grabias et al. [15] believed that curettage or biopsy of may predispose the bone to a pathological fracture, and there is no accurate indication of the success of curettage and bone grafting. Thus, an incidental lesion that suggests the need for biopsy should be dealt with caution as curettage and biopsy without internal fixation might lead to a frank fracture.

This being a retrospective study, we did not have data on preoperative hip scores, which is a limitation of our study. Also, we would have preferred to have a longer follow-up.

## Conclusion

Shepherd’s crook deformity can be treated by osteotomy; fixation with intramedullary implants with neck cross-pinning is a preferable method of treating shepherd crook deformity. For pathological fractures, fixation with an intramedullary nail provides reliable fixation and stability even though it might not be accompanied by complete resolution of the fibrodysplastic lesion. More than one procedure may be required. The senior author’s preference is load sharing IM reconstruction nail with or without osteotomies as needed to address the femoral deformities.

Incidental or asymptomatic lesions should not be treated especially with curettage alone. The role of curettage and grafting with allograft, autograft or calcium sulfate is questionable.
